# Prevalence of *Chlamydia trachomatis* in Pregnant Iranian Women: A Systematic Review and Meta-Analysis

**DOI:** 10.22074/ijfs.2018.5191

**Published:** 2018-06-20

**Authors:** Milad Azami, Gholamreza Badfar, Akram Mansouri, Mohammad Hossein Yekta Kooshali, Wesam Kooti, Zeinab Tardeh, Ali Soleymani, Shamsi Abbasalizadeh

**Affiliations:** 1 Student Research Committee, Ilam University of Medical Sciences, Ilam, Iran; 2 Women’s Reproductive Health Research Center, Tabriz University of Medical Sciences, Tabriz, Iran; 3 Department of Pediatrics, Behbahan Faculty of Medical Sciences, Behbahan, Iran; 4 School of Nursing and Midwifery, Ahvaz Jundishapour University of Medical Sciences, Ahvaz, Iran; 5 Student Research Committee, School of Nursing, Midwifery, and Paramedicine, Guilan University of Medical Sciences, Rasht, Iran; 6 Student Research Committee, Kurdistan University of Medical Sciences, Sanandaj, Iran; 7 Dezful University of Medical Sciences, Dezful, Iran

**Keywords:** *Chlamydia trachomatis*, Meta-Analysis, *Mycoplasma Hominis*, Pregnant Women, *Ureaplasma Urealyticum*

## Abstract

Several studies have been conducted regarding the prevalence of *Chlamydia trachomatis*, *Mycoplasma hominis*, and Ureaplasma urealyticum in pregnant Iranian women. However, it is necessary to combine the previous results to present a general assessment. We conducted the present study based on systematic review and meta-analysis studies according to the Preferred Reporting Items for Systematic Reviews and Meta-Analyses (PRISMA). We searched the national and international online databases of MagIran, IranMedex, SID, MedLib, IranDoc, Scopus, PubMed, ISI Web of Knowledge, and Google Scholar search engine for certain MeSH keywords until June 16, 2017. In addition, heterogeneity, sensitivity analysis, subgroup analysis, and publication bias were performed. The data were analyzed using random-effects model and Comprehensive Meta-Analysis version 2 and P value was considered lower than 0.05. The prevalence of *Chlamydia trachomatis* in 11 surveyed articles that assessed 2864 pregnant Iranian women was 8.74% [95% confidence interval (CI): 5.40-13.84]. The prevalence of *Chlamydia trachomatis* was estimated 5.73% (95% CI: 2.09-14.73) and 13.55% (95% CI: 11.23-16.25) by enzyme-linked immunosorbent assay (ELISA) and polymerase chain reaction (PCR), respectively which the difference was not significant (P=0.082). The lowest and highest prevalence of *Chlamydia trachomatis* was estimated in Tehran province [4.96% (95% CI: 2.45-9.810)] and Ardabil province [28.60% (95% CI: 20.61-38.20)], respectively. This difference was statistically significant (P<0.001). Meta-regression for the prevalence of *Chlamydia trachomatis* based on year of the studies was significant with increasing slope (P=0.017). According to the systematic review, the prevalence of *Mycoplasma hominis* and Urea plasma urealyticum indicated 2 to 22.8% (from 4 articles) and 9.1 to 19.8% (from 3 articles), respectively. There was no evidence of publication bias (P value for Begg and Eggers’ tests was 0.161 and 0.173, respectively). The prevalence of *Chlamydia trachomatis* is high among pregnant Iranian women. Screening pregnant women as part of preventive measures seem necessary considering the potential for maternal and fetal complications.

## Introduction

Pregnancy is a serious period in women´s lives, which is related to physiological changes, such as weakening the immune system (1, 2). Reproductive tract infections are one of the most serious public health issues in developed and developing countries (3). *Chlamydia trachomatis* is one of the most common sexually transmitted diseases worldwide (4). Colonization of *Chlamydia trachomatis* in the reproductive tract of pregnant women causes complications such as infertility, chronic pelvic pain, ectopic pregnancy, premature rupture of membranes (PROM), prematurity, spontaneous abortion, and perinatal mortality (5, 6). The prevalence of *Chlamydia trachomatis* infection is currently increasing throughout the world. The treatment costs of *Chlamydia trachomatis* infection is estimated to be more than 2 million US dollars. Diagnostic costs are much lower than treatment costs. Therefore, timely diagnosis and screening can decrease the prevalence of reproductive tract infections and reduce treatment costs of this disease (7).

 The level of immunity in the body decreases during pregnancy (2). A weak immune system increases the risk factor for the entrance of infectious agents into the vagina. *Ureaplasma urealyticum* and *Mycoplasma hominis* are genital mycoplasmas that can be detected in the lower genitourinary tract of sexually active women as a result of colonization of the genital tract through sexual contact (5). These microorganisms can affect each part of the urogenital system and cause infection (8).

These microorganisms have an important role in infections and potential complications during pregnancy. Therefore, it is necessary to be aware of prevalence patterns to plan and screen pregnant women for these microorganisms. Several studies have been conducted in Iran to determine the prevalence of *Chlamydia trachomatis*, *Mycoplasma hominis*, and *Ureaplasma urealyticum* in pregnant Iranian women (9-21). Combining the previous results to present a general assessment seems necessary. A review of all relevant documents and presenting a general assessment based on systematic review and meta-analysis studies can provide a more detailed picture of the dimensions of this problem in pregnant women (22-24). Therefore, we have conducted the present meta-analysis women from Iran.

## Materials and Methods

### Study protocol

We conducted the present study based on systematic review and meta-analysis studies according to the Preferred Reporting Items for Systematic Reviews and Meta-Analyses (PRISMA) (24). To avoid bias, two researchers conducted independent searches, selection of studies, quality assessment, and data extraction. In case of dispute, the case was referred to a third researcher. The final agreement was reached as a general discussion.

### Search strategy

We searched national online databases such as MagIran, IranMedex, SID, MedLib, and IranDoc, in addition to the international databases Scopus, PubMed, ISI Web of Knowledge, and Google Scholar search engine till June 16, 2017. To maximize the comprehensiveness of the search, we used MeSH keywords with all possible combinations with “OR” and “AND” in the English databases: 'Epidemiology', 'Prevalence', 'Chlamydia', 'Ureaplasma', 'Mycoplasma', 'Sexually transmitted diseases', 'Reproductive tract infections', 'Pregnant women', 'Pregnancy', 'Gestational', and 'Iran'. At the end of the search, the titles of the collected articles were entered into EndNote™ software to find similar articles.

### The studied population

The studied population included pregnant Iranian women. The positive result for *Chlamydia trachomatis* was determined by enzyme-linked immunosorbent assay (ELISA) or polymerase chain reaction (PCR). The positive results for *Mycoplasma hominis* and *Ureaplasma urealyticum* were determined by PCR (25, 26).

### Inclusion and exclusion criteria

Inclusion criteria of this study consisted of a reference to the prevalence of *Chlamydia trachomatis*, *Mycoplasma hominis*, and *Ureaplasma urealyticum* in pregnant Iranian women, either in Persian or English. Exclusion criteria were: non-random sample size; irrelevance; limited information such as failure to report disease prevalence; review articles, case reports, and editorials; duplicate articles; and failure to diagnose based on laboratory results.

### Quality assessment

In the next step, researchers assessed the quality of articles according to the modified Newcastle Ottawa Scale (NOS) for cross-sectional studies (27) that consisted of 8 sections in 4 categories, including selection, comparability, exposure assessment, and outcome. This scale ranges from 0 to 9 point. The minimum acceptable score was 7.

### Data extraction

 All included articles were prepared for data extraction by a pre-prepared checklist. The checklist included the author’s name, year of the study, the location of the study, study design, sample volume, mean age, quality score and the prevalence of *Chlamydia trachomatis*, *Mycoplasma hominis*, and *Ureaplasma urealyticum*.

### Statistical analysis 

The variance of each study was estimated according to the binomial distribution. We used the Q test and I^2^ index to assess the heterogeneity of the studies (28). Studies with heterogeneity greater than 75% fell into the category of high heterogeneity. If the I^2^ index was lower than 25%, the heterogeneity was low; between 25-75% indicated medium heterogeneity, and higher than 75% indicated high heterogeneity. Due to the significance of the I^2^ index, we used the random effects model for the meta-analysis (29, 30). Sensitivity analysis was conducted by deleting every single study from meta-analysis. Subgroup analysis based on province, diagnostic test, year of studies and meta-regression based on years and diagnostic test were used to detect heterogeneity of papers with the subject of *Chlamydia trachomatis*. Egger and Beggs’ tests were used to assess publication bias. The data were analyzed using Comprehensive Meta-Analysis (CMA) version 2. P values were considered less than 0.05.

## Results

### Search results and characteristics of the eligible studies

**Fig.1:**
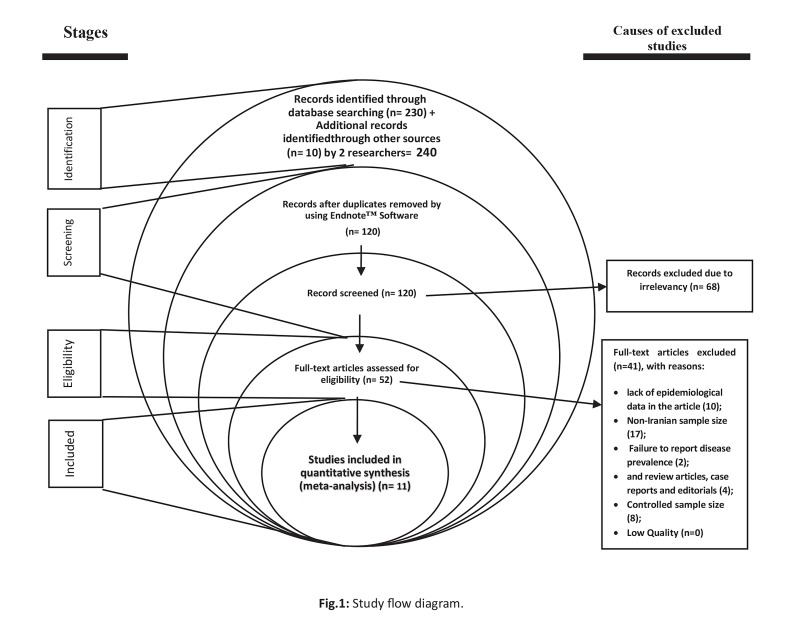
Study flow diagram.

We located 240 relevant studies in the systematic review. There were 229 studies omitted due to the following reasons: duplicate studies [120]; irrelevance [68]; lack of epidemiological data in the article [10]; non-Iranian sample size [17]; failure to report disease prevalence [2]; controlled sample size [8]; and review articles, case reports and editorials [4] (Fig .1). Finally, 11 qualified studies for *Chlamydia trachomatis* (9-16, 20, 21) entered the meta-analysis process. In addition, 4 and 3 qualified studies for *Mycoplasma hominis* (17-20) and *Ureaplasma urealyticum* (16-18), respectively entered the systematic review process (Table 1). The mean age of the pregnant women belong to the qualified studies was estimated 27.45 years old (95% CI: 26.03-28.88).

**Table 1:** Characteristics of 13 studies on *Chlamydia trachomatis*, *Mycoplasma hominis*, and *Ureaplasma urealyticum* in pregnant Iranian women


Reference	First author	Place	Year	Sample size	Prevalence (%)			Test
					*Chlamydia trachomatis*	*Mycoplasma hominis*	*Ureaplasma urealyticum*		

(9)	Sohrabi et al.	Ahwaz	2005	79	10.1			ELISA
(10)	Rashidi et al.	Tehran	2008	225	11.1			PCR
(11)	Khezerdoust et al.	Tehran	2006	667	3.3			ELISA
(11)	Khezerdoust et al.	Tehran	2006	447	2.2			ELISA
(12)	Chamani Tabriz et al.	Tehran	2003	340	11.2			PCR
(13)	Behrozi and Badamee	Tehran	1994	400	2.75			ELISA
(14)	Ahmadi et al.	Sanandaj	2012	218	17.43			PCR
(15)	Mobasheri et al.	Ardal	2010	85	4.7		19.8	ELISA
(16)	Rohi et al.	Ardabil	2010	100	28.6		15	PCR
(17)	Sobouti et al.	Tehran	2010	165		15	9.1	PCR
(18)	Azizmohammadi et al.	Tehran	2015	350		2.8		PCR
(19)	Mohseni et al.	Tonekabon	2012	44		22.7		PCR
(20)	Haghighi Hasanabad et al.	Sabzevar	2010	196	14.8	2.04		PCR
(21)	Sisakht et al.	Yasuj	2010	107	14.02			PCR


### Prevalence of *Chlamydia trachomatis*

**Fig.2:**
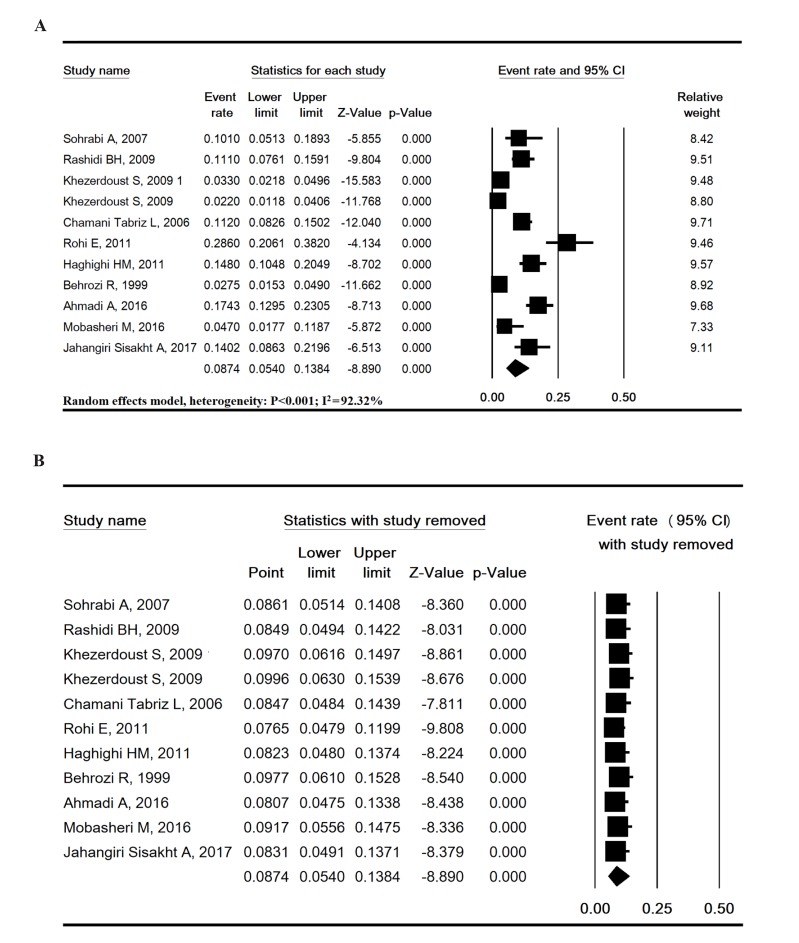
Forest plot. Prevalence of *Chlamydia trachomatis* in pregnant Iranian women. A. Overall estimate and B. Sensitivity analysis.

We assessed 11 surveyed articles that had a sample size of 2864 pregnant Iranian women and determined the prevalence to be 8.74% (95% CI: 5.40-13.84), and high heterogeneity was estimated between studies (P<0.001, I^2^ = 92.32%) for *Chlamydia trachomatis* (Fig .2A). The lowest prevalence pertained to the study by Khezerdoust et al. (11) in Tehran (2.2%), whereas the highest prevalence was reported by Rohi et al. (16) in Ardebil (28.6%). Sensitivity analysis indicated that the pooled results were robust (Fig .2B).

### Subgroup analysis of *Chlamydia trachomatis* prevalence based on diagnostic test, year of studies and province

The prevalence of *Chlamydia trachomatis* by enzyme-linked immunosorbent assay (ELISA) was 5.73% (95% CI: 2.09-14.73), and for polymerase chain reaction (PCR) it was 13.55% (95% CI: 11.23-16.25). The difference was not significant (P=0.082). The prevalence of *Chlamydia trachomatis* sub-grouped by year of study (2005 to 2009 versus 2010 to 2014) was statistically significant (P=0.016, Table 2). The prevalence of *Chlamydia trachomatis* was estimated based on the province, and the lowest prevalence was estimated in Tehran province [4.96% (95% CI: 2.45-9.81)] whereas the highest prevalence estimated in Ardebil province [28.60% (95% CI: 20.61-38.20)]. This difference was statistically significant (P<0.001, Table 2).

### Meta-regression for the prevalence of *Chlamydia trachomatis*

**Fig.3:**
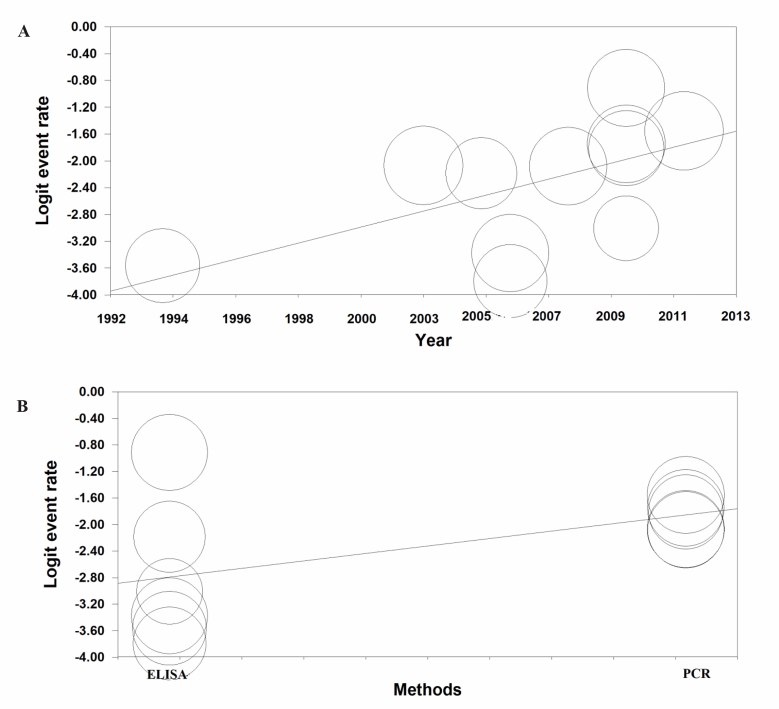
Meta-regression of prevalence of *Chlamydia trachomatis* in pregnant women. A. Based on year of studies and B. Based on diagnostic test (Larger circles indicate larger sample size).

Meta-regression for the prevalence of *Chlamydia trachomatis* based on year of studies was significant (meta-regression coefficient: 0.110, 95% CI: 0.019-0.201, P=0.017) and also based on diagnostic test was not significant (meta-regression coefficient: 0.093, 95% CI:-0.038-1.910, P=0.059) (Fig .3).

**Table 2:** Prevalence of *Chlamydia trachomatis* in pregnant women in Iran according to diagnostic test, year of studies and province


Variable		Studies (n)	Sample size (n)	Prevalence (%)	95% CI	I^2^ (%)	P value (heterogeneity)

Diagnostic test	ELISA	6	1778	5.73	2.09-14.73	94.81	<0.001
	PCR	5	1086	13.55	11.23-16.25	31.11	0.214
	Subgroup differences: Q value=3.029, df=1, P=0.082						
Year of studies	2005-2009	4	1418	5.41	2.40-11.73	90.25	<0.001
	2010-2014	5	706	15.67	10.47-22.78	78.05	0.001
	Subgroup differences: : Q value=5.76, df=1, P=0.016						
Province	Khuzestan	1	79	10.10	5.13-18.93	-	-
	Tehran	5	2079	4.96	2.45-9.81	92.19	<0.001
	Ardebil	1	100	28.60	20.61-38.20	-	-
	Razavi Khorasan	1	196	14.8	10.48-20.49	-	-
	Kurdistan	1	218	17.43	12.95-23.05	-	-
	Chaharmahal and Bakhtiari	1	85	4.7	1.77-11.87	-	-
	Kohgiloyeh and Boyerahmad	1	107	14.02	8.63-21.96	-	-
	Subgroup differences: : Q value=32.88, df=6, P<0.001						


CI; Confidence interval, I^2^; Heterogeneity in Meta-analysis, ELISA; Enzyme-linked immunosorbent assay, and PCR; Polymerase chain reaction.

**Fig.4:**
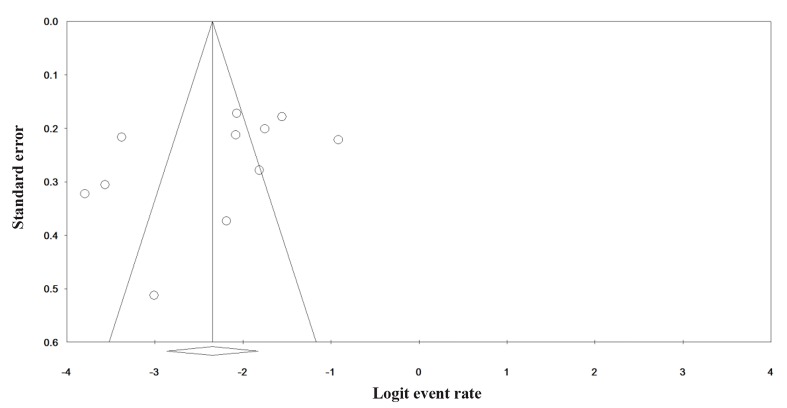
Publication bias in the studies for the prevalence of *Chlamydia trachomatis*.

### Prevalence of *Mycoplasma hominis* and *Ureaplasma urealyticum*

The systematic review results of *Mycoplasma hominis* and *Ureaplasma urealyticum* indicated the prevalence of 2% to 22.8% (from 4 articles) (17-20) and 9.1 to 19.8% (from 3 articles) (16-18), respectively.

### Publication bias

Funnel plot for the prevalence of *Chlamydia trachomatis* did not reveal significant publication bias (P value for Begg and Eggers’ tests was 0.161 and 0.173, respectively) (Fig .4).

## Discussion

Awareness of the prevalence pattern and screening for diseases that affect the health of the mother and fetus is necessary during pregnancy. The present study is the first systematic review to assess the prevalence of *Chlamydia trachomatis*, *Mycoplasma hominis*, and *Ureaplasma urealyticum* in pregnant Iranian women.

We have determined the prevalence of *Chlamydia trachomatis* in 11 surveyed articles that had a sample size of 2864 pregnant Iranian women to be 8.74% (95% CI: 5.40-13.84). The prevalence of this disease is 10.1% in China (31), 10.5% in Saudi Arabia (32), and 35% in India (33). However, the results of the present study are similar to those reported in Scotland (34).

The prevalence of *Chlamydia trachomatis* in some studies was higher than the present study (35, 36). On the other hand, the results of other studies were in the same range as the present study (37, 38), though not exactly identical. Inconsistent results with the present study might be due to cultural differences, social and religious norms, and mean age of the studied populations. The high prevalence of this infection in women age 20 and above might be due to early onset of sexual intercourse, numerous pregnancies, and the use of oral contraceptives (39, 40). Women with vaginal secretions and inflammatory changes in cervical cytology are more prone to infection and should be examined by their gynecologists (41). Several documents have demonstrated that dysuria, vaginal discharge, and lower abdominal pain may be clinical symptoms of this infection (41, 42), which are more common in pregnant women.

Age (particularly 18-27 years) and socioeconomic conditions such as an urban residence or low income (43) are among the risk factors for *Chlamydia trachomatis* in pregnant women. A study in Japan has reported a significantly high prevalence of *Chlamydia trachomatis* in primiparous pregnant women (44).

Recent studies report a significant relationship between *Chlamydia trachomatis* infection to preterm delivery (5, 45). The importance of *Chlamydia trachomatis* for midwives is due to the ability of this microorganism to cause urethritis, cervicitis, preterm births, PROM, and neonatal infections as the baby passes through the birth canal, in addition to abortion, maternal mortality, and stillbirth. Repeated screening tests in the first prenatal examination and during the third trimester of pregnancy, along with successful treatment with erythromycin can reduce the complications of pregnancy according to the American College of Obstetricians and Gynecologists (5, 6, 45, 46).

These bacteria can be easily detected by cell culture and serological methods that use micro-immunofluorescence techniques, ELISA, the complement fixation test (CFT), antigen detection methods, molecular methods (DNA hybridization, nucleic acid amplification techniques), and direct cytological methods (Giemsa, Gimenez, and hematoxylin stains) (25, 26). The best and most cost-effective method to determine whether the infection during pregnancy is acute or chronic the ELISA test (47, 48). In the present study, we have noted that the prevalence of *Chlamydia trachomatis* according to PCR results was not significantly more than ELISA (P=0.082).

The prevalence of *Mycoplasma hominis* in pregnant Iranian women from 4 studies was 2 to 22.8% (17-20). The prevalence of *Ureaplasma urealyticum* in Iranian pregnant women was 9.1 to 19.8% (16-18). Meta-analysis was not performed on the prevalence of *Mycoplasma hominis* and *Ureaplasma urealyticum* because of a scanty number of studies. Therefore, we recommend conducting more research in this area for future studies. *Mycoplasma hominis* had the following prevalence in other countries: 3.7% (Poland), 31.5% (Portugal), 11.2% (Japan). *Ureaplasma urealyticum* had the following prevalence in other countries: 29.8% (Poland), 27.8% (Portugal), and 8.7% (Japan) (49-51). These results did not agree with the present study, which might be due to cultural differences, social and religious norms, and the mean age of the studied populations.

*Mycoplasma hominis* is isolated from the vaginal secretions of 15-70% of women. *Ureaplasma urealyticum*is is isolated from the vaginal secretions of 40-95% of women (8, 52-56). *Mycoplasma hominis* and *Ureaplasma urealyticum*is are transferred to the fetus during pregnancy or normal vaginal delivery. They are often associated with cervicitis, vaginitis, pyelonephritis, pelvic inflammatory disease, postpartum septicemia, uterine infections, meningitis, PROM, postpartum fever, preterm delivery, and low weight premature birth (8, 57, 58). Most premature births for women with these two infections happen before the 34th week of pregnancy (59, 60). However, no significant relationship has been found between these infections and adverse effects on pregnancy in some studies (51). Studies conducted on *Mycoplasma hominis* have demonstrated that it caused adnexal lesions but not salpingitis (53). *Ureaplasma urealyticum* is the main cause of non chlamydial and nongonococcal urethritis, chorioamnionitis, cervicitis, vaginitis, sepsis and preterm delivery. Moreover, it may cause pneumonia, meningitis and even death of the infant as the baby passes through the birth canal (56). The role of *Ureaplasma urealyticum* has not been specified (54). PCR is often used to diagnose this infection in Iran. However, the molecular technique has also been used in some studies in Iran (61). Therefore, considering the fact that failure to diagnose, prevent, and treat these infections leads to dangerous complications, it is necessary to identify these bacteria, particularly in pregnant women (53, 62).

Factors that increase the prevalence of prenatal infections in women include young age (adolescents and young adults); use of an intrauterine device (IUD); low level of education, unemployment, and low income; multiple sex partners; not using a condom, diaphragm or spermicide; lack of attention to individual health care for both men; and women and smoking, alcohol consumption, and drugs (25, 63, 64).

Several meta-analysis studies in Iran have focused on other infections in pregnant women and reported the following results: prevalence of urinary tract infection (11.2%) (65), hepatitis B (2%) (66, 67), and Helicobacter pylori (45.9%) (68, 69). According to Ahmadi et al. (70), the prevalence of urogenital mycoplasmas in the male population was 11.1% (95% CI: 7.4-16.4) and 12.8% (95% CI: 9.8-16.5) in females, which was high. Hence, *Chlamydia trachomatis* is one of the most common infections in pregnant Iranian women. Determining the causes of these infections and methods of prevention should be among the medical priorities for pregnant Iranian women to ensure the health of the next generation.

The limitations of the study included the failure to search using a combination of words in internal databases due to low sensitivity and the inability to perform further subgroup analysis because of the limited number of studies.

Future case-control studies to determine the role of various risk factors in Iranian societies seems necessary.

## Conclusion

The high prevalence of reproductive tract infections among pregnant Iranian women necessitates screening these women as a preventive measure. Therefore, timely recognition and treatment of this disease can prevent maternal and fetal complications.

## References

[1] Azami M, Darvishi Z, Sayehmiri K (2016). Systematic review and meta-analysis of the prevalence of anemia among pregnant Iranian women (2005-2015). Shiraz E-Med J.

[2] Workowski KA, Levine WC, Wasserheit JN (2002). Centers for Disease Control and Prevention, Atlanta, Georgia.U.S.Centers for Disease Control and Prevention guidelines for the treatment of sexually transmitted diseases: an opportunity to unify clinical and public health practice. Ann Intern Med.

[3] Kamara P, Hylton-Kong T, Brathwaite A, Del Rosario GR, Kristensen S, Patrick N (2000). Vaginal infections in pregnant women in Jamaica: prevalence and risk factors. Int J STD AIDS.

[4] Joseph Davey DL, Shull HI, Billings JD, Wang D, Adachi K, Klausner JD (2016). Prevalence of curable sexually transmitted infections in pregnant women in low- and middle-income countries from 2010 to 2015: a systematic review. Sex Transm Dis.

[5] Capoccia R, Greub G, Baud D (2013). Ureaplasma urealyticum, Myco¬plasma hominis and adverse pregnancy outcomes. Curr Opin Infect Dis.

[6] Adachi K, Nielsen-Saines K, Klausner JD (2016). Chlamydia trachomatis infection in pregnancy: the global challenge of preventing adverse pregnancy and infant outcomes in Sub-Saharan Africa and Asia. Biomed Res Int.

[7] Schachter J, Stamm WE (1983). Chlamydia trachomatis.International perspectives on neglected STD's.New York.McGraw-Hill Book Co.

[8] Bjartling C, Osser S, Persson K (2012). Mycoplasma genitalium in cervicitis and pelvic inflammatory disease among women at a gynecologic outpatient service.Am J Obstet Gynecol.

[9] Sohrabi A, Samarbafzadeh AR, Makvandi M, Maraghi Sh, Razi T, Darban D (2007). A seroepidemiological study of Parvovirus B19, Toxoplasma gondii and Chlamydia trachomatis in pregnant women referring to Obs & Gyn ward of Ahwaz Imam Khomeini Hospital. J Reprod Infertil.

[10] Rashidi BH, Tabriz L, Haghollahi F, Ramezanzadeh F, Shariat M, Foroushani AR (2009). Prevalence of Chlamydia trachomatis infection in fertile and infertile women; a molecular and serological study. J Reprod Infertil.

[11] Khezerdoust S, Haghollahi F, Roostaie S, Badami N, Naghizadeh MM, Jafarabadi M (2009). Chlamydia trachomatis infection in pregnant women. J Reprod Infertil.

[12] Chamani Tabriz L, Jeddi Tehrani M, Mosavi Jarrahi A, Zeraati H, Ghasemi J, Asgari S (2006). The prevalence of Chlamydia trachomatis infection by molecular analysis of urine samples in women attending OB & GYN clinics in Tehran. J Reprod Infertil.

[13] Behrozi R, Badamee N (1999). The prevalence of chlamydial infection in pregnant women in hospitals of Tehran University of Medical Sciences in 1994 (a pre-test study). J Mazandaran Univ Med Sci.

[14] Ahmadi A, Khodabandehloo M, Ramazanzadeh R, Farhadifar F, Roshani D, Ghaderi E (2016). The relationship between Chlamydia trachomatis genital infection and spontaneous abortion. J Reprod Infertil.

[15] Mobasheri M, Saeedi Varnamkhast N, Karimi A, Banaeiyan S (2014). Prevalence study of genital tract infections in pregnant women referred to health centers in Iran. Turk J Med Sci.

[16] Rohi E, Ghasemi K, Kahnemoii Agdam F (2011). Incidence of Non-gonococcal infection in childbearing and pregnant women in Ardabil. International Journal of Molecular and Clinical Microbiology.

[17] Sobouti B, Fallah S, Mobayen M, Noorbakhsh S, Ghavami Y (2014). Colonization of Mycoplasma hominis and Ureaplasma urealyticum in pregnant women and their transmission to offspring. Iran J Microbiol.

[18] Azizmohammadi S, Azizmohammadi S (2015). Antimicrobial susceptibility patterns of Ureaplasma urealyticum and Mycoplasma hominis isolated from pregnant women. Iran Red Crescent Med J.

[19] Mohseni R, Sadeghi F, Mirinargesi M, Eghbali M, Dezhkame S, Ghane M (2013). A study on the frequency of vaginal species of Mycoplasma genitalium, Gardnerella vaginalis and Neisseria gonorrhoeae among pregnant women by PCR technique. Int J Mol Clin Microbiol.

[20] Haghighi Hasanabad M, Mohammadzadeh M, Bahador A, Fazel N, Rakhshani H, Majnooni A (2011). Prevalence of Chlamydia trachomatis and Mycoplasma genitalium in pregnant women of Sabzevar-Iran. Iran J Microbiol.

[21] Sisakht AJ, Omidifar N, Mohamadkhani N, Karimpoorfard M, Kargar M, Shokripour M (2017). Assessing the presence of Chlamydia trachomatis genome in pregnant women with spontaneous abortion using polymerase chain reaction method in Yasuj: first report from Southwest of Iran. J Educ Health Promot.

[22] Sayehmiri K, Tavan H, Sayehmiri F, Mohammadi I, V. Carson K (2014). Prevalence of epilepsy in Iran: a meta-analysis and systematic review. Iran J Child Neurol.

[23] Sayehmiri K, Abangah G, Kalvandi G, Tavan H, Aazami S (2018). Prevalence of peptic ulcer in Iran: Systematic review and meta-analysis methods. J Res Med Sci.

[24] Moher D, Liberati A, Tetzlaff J, Altman DG (2009). PRISMA Group.Preferred reporting items for systematic reviews and meta-analyses: the PRISMA statement. PLoS Med.

[25] Sweet RL (2012). Pelvic inflammatory disease: current concepts of diagnosis and management. Curr Infect Dis Rep.

[26] Amirmozafari N, Forohesh H, Ganji L (2007). Comparison of Microimmunofluorescence , ELISA, Rapid Detection Kit(DIMA) and Gimenez Staining for Detection of Chlamydial Induced Cervicitis. Razi Journal of Medical Sciences.

[27] Stang A (2010). Critical evaluation of the Newcastle-Ottawa scale for the assessment of the quality of nonrandomized studies in meta-analyses. Eur J Epidemiol.

[28] Higgins JP, Green S (2011). Cochrane handbook for systematic reviews of interventions.Hoboken, NJ, USA.John Wiley & Sons.

[29] Ades AE, Lu G, Higgins JP (2005). the interpretation of random effects meta-analysis in decision models. Med Decis Making.

[30] Higgins JP, Thompson SG, Deeks JJ, Altman DG (2003). Measuring inconsistency in meta-analyses. BMJ.

[31] Chen XS, Yin YP, Chen LP, Thuy NT, Zhang GY, Shi MQ (2006). Sexually transmitted infections amongpregnant women attending an antenatal clinic in Fuzhou, China. Sex Transm Dis.

[32] Alzahrani AJ, Obeid OE, Hassan MI, Almulhim AA (2010). Screening of pregnant women attending the antenatalcare clinic of a tertiary hospital in eastern Saudi Arabiafor Chlamydia trachomatis and Neisseria gonorrhoeae infections. Indian J Sex Transm Dis.

[33] Sharma M, Sethi S, Daftari S, Malhotra S (2003). Evidence of chlamydial infection in infertile women with fallopian tube obstruction. Indian J Pathol Microbiol.

[34] Borges da Costa J, Azevedo J, Santo I (2010). Sexually transmitted infections and related sociodemographicfactors in Lisbon’s major venereology clinic: adescriptive study of the first 4 months of 2007. J EurAcad Dermatol Venereol.

[35] Mudau M, Peters RP, De Vos L, Olivier DH, J Davey D, Mkwanazi ES (2017). High prevalence of asymptomatic sexually transmitted infections among human immunodeficiency virus-infected pregnant women in a low-income South African community. Int J STD AIDS.

[36] Higgins SP, Klapper PE, Struthers JK, Bailey AS, Gough AP, Moore R (1998). Detection of male genital infection with Chlamydia trachomatis and Neisseria gonorrhoeae using an automated multiplex PCR system (Cobas Amplicor). Int J STD AIDS.

[37] Silveira MF, Sclowitz IK, Entiauspe LG, Mesenburg MA, Stauffert D, Bicca GL (2017). Chlamydia trachomatis infection in young pregnant women in Southern Brazil: a cross-sectional study. Cad Saude Publica.

[38] Schönfeld A, Feldt T, Tufa TB, Orth HM, Fuchs A, Mesfun MG (2018). Prevalence and impact of sexually transmitted infections in pregnant women in central Ethiopia. Int J STD AIDS.

[39] Paavonen, J (1992). Genital Chlamydia trachomatis infection in the female. J Infect.

[40] Betha K, Robertson JM, Tang G, Haggerty CL (2016). Prevalence of Chlamydia trachomatis among childbearing age women in India: a systematic review. Infect Dis Obstet Gynecol.

[41] Carrington D, Ridgway GL (1992). Chlamydia. J Infect.

[42] Nenoff P, Manos A, Ehrhard I, Krüger C, Paasch U, Helmbold P (2017). Non-viral sexually transmitted infections-epidemiology, clinical manifestations, diagnostics and therapy: part 2: Chlamydia and mycoplasma. Hautarzt.

[43] Singh V, Rastogi S, Garg S, Kapur S, Kumar A, Salhan S (2002). Polymerase chain reaction for detection of endocervical Chlamydia trachomatis infection in women attending a gynecology outpatient department in India. Acta Cytol.

[44] Shimano S, Nishikawa A, Sonoda T, Kudo R (2004). Analysis of the prevalence of bacterial vaginosis and Chlamydia trachomatis infection in 6083 pregnant women at a hospital in Otaru, Japan. J Obstet Gynaecol Res.

[45] O’Higgins A, Jackson V, Lawless M, Le Blanc D, Connolly G, Drew R (2017). Screening for asymptomatic urogenital Chlamydia trachomatis infection at a large Dublin maternity hospital: results of a pilot study. Ir J Med Sci.

[46] Gaydos CA, Jett-Goheen M, Barnes M, Dize L, Barnes P, Hsieh YH (2016). Use of a risk quiz to predict infection for sexually transmitted infections: a retrospective analysis of acceptability and positivity. Sex Transm Infect.

[47] (1991). Pelvic inflammatory disease: guidelines for prevention and management. MMWR Recomm Rep.

[48] Sweat M, Gregorich S, Sangiwa G, Furlonge C, Balmer D, Kamenga C (2000). Cost-effectiveness of voluntary HIV-1 counselling and testing in reducing sexual transmission of HIV-1 in Kenya and Tanzania. Lancet.

[49] Zdrodowska-Stefanow B, Klosowska WM, Ostaszewska-Puchalska I, Bulhak-Koziol V, Kotowicz B (2006). Ureaplasma urealyticum and Mycoplasma hominis infection in women with urogenital diseases. Adv Med Sci.

[50] Domingues D, Távora Tavira L, Duarte A, Sanca A, Prieto E, Exposto F (2003). Genital mycoplasmas in women attending a family planning clinic in Guine-Bissau and their susceptibility to antimicrobial agents. Acta tropica.

[51] Kataoka S, Yamada T, Chou K, Nishida R, Morikawa M, Minami M (2006). Association between preterm birth and vaginal colonization by mycoplasmas in early pregnancy. J Clin Microbiol.

[52] Oakeshott P, Aghaizu A, Hay P, Reid F, Kerry S, Atherton H (2010). Is Mycoplasma genitalium in women the “new chlamydia?.” a community-based prospective cohort study. Clin Infect Dis.

[53] McGowin CL, Anderson-Smits C (2011). Mycoplasma genitalium: An emerging cause of sexually transmitted disease in women. PLoS Pathog.

[54] Shahshahan Z, Hoseini N (2012). Investigating prevalence of mycoplasma and ureaplasma infection in pregnant women with preterm labor. The Iranian Journal of Obstetrics, Gynecology and Infertility.

[55] Amirmozafari N, Jeddi F, Masjedian F, Haghighi L (2009). Prevalence of Mycoplasma hominis and Ureaplasma urealyticum in Genital Tract Infections. Razi Journal of Medical Sciences.

[56] Ramezani A, Nazguiy F, Mafakheri M (2002). Prevalence of Mycoplasma and Ureaplasma hemlyiticom in patients with septic abortion admitted to a private clinic in 2001. Iran Infectious and Tropical Diseases Journal.

[57] Schlicht MJ, Lovrich SD, Sartin JS, Karpinsky P, Callister SM, Agger WA (2004). High prevalence of genital mycoplasmas among sexually active young adults with urethritis or cervicitis symputoms in La Crosse, Wisconsin. J Clin Mictobiol.

[58] Payne MS, Goss KC, Connett GJ, Kollamparambil T, Legg JP, Thwaites R (2010). Molecular microbiological characterization of preterm neonates at risk of bronchopulmonary dysplasia. Pediatr Res.

[59] Usui R, Ohkuchi A, Matsubara S, Izumi A, Watanabe T, Suzuki M (2002). Vaginal lactobacilli and preterm birth. J Perinat Med.

[60] Goldenberg RL, Andrews WW, Goepfert AR, Faye-Petersen O, Cliver SP, Carlo WA (2008). The alabama preterm birth study: umbilical cord blood Ureaplasma urealyticum and Mycoplasma hominis cultures in very preterm newborn infants. Am J Obstet Gynecol.

[61] Mirnejad R, Amirmozafari N, Kazemi B (2010). Molecular identification and genotyping of Mycoplasma genitalium in women with genital infections by PCR-RFLP. Iran J Med Microbiol.

[62] Short VL, Totten PA, Ness RB, Astete SG, Kelsey SF, Haggerty CL (2009). Clinical presentation of Mycoplasma genitalium Infection versus Neisseria gonorrhoeae infection among women with pelvic inflammatory disease. Clin Infect Dis.

[63] Kheiyrkhah M, Asadzadeh F, Farshad MM (2012). Incidence of symptoms and complications of pelvic inflammatory disease. Journal of Health and Care.

[64] Abdollahiyan P, Shodjai Tehrani H, Asghri Sh, Oudi M (2005). Relative frequency of gonococcal endocervisitis and some associated factors in reproductive age women. J Guilan Univ Med Scis.

[65] Ghafari M, Baigi V, Cheraghi Z, Doosti-Irani A (2016). The prevalence of asymptomatic bacteriuria in Iranian pregnant women: a systematic review and meta-analysis. PLoS One.

[66] Azami M, Khataee M, Bigam Bigdeli Shamlo M, Abasalizadeh F, Rahmati Sh, Abasalizadeh Sh (2016). Prevalence and Risk factors of hepatitis B Infection in pregnant women of Iran: a systematic review and meta-analysis. The Iranian Journal of Obstetrics, Gynecology and Infertility.

[67] Badfar G, Shohani M, Nasirkandy MP, Mansouri A, Abangah G, Rahmati S (2018). Epidemiology of hepatitis B in pregnant Iranian women: a systematic review and meta-analysis. Arch Virol.

[68] Abbasalizadeh Sh, Darvishi Z, Abbasalizadeh F, Azami M, Borji M, Afshar Safavid A (2016). The prevalence of helicobacter pylori infection among Iranian pregnant women-a meta-analysis study. Journal of Knowledge & Health.

[69] Azami M, Parizad Nasirkandy M, Mansouri A, Darvishi Z, Rahmati Sh, Abangah Gh (2017). Global prevalence of helicobacter pylori infection in pregnant women: a systematic review and meta-analysis study. Int J Women's Health Reprod Sci.

[70] Ahmadi MH, Mirsalehian A, Bahador A (2016). Prevalence of urogenital mycoplasmas in iran and their effects on fertility potential: a systematic review and meta-analysis. Iran J Public Health.

